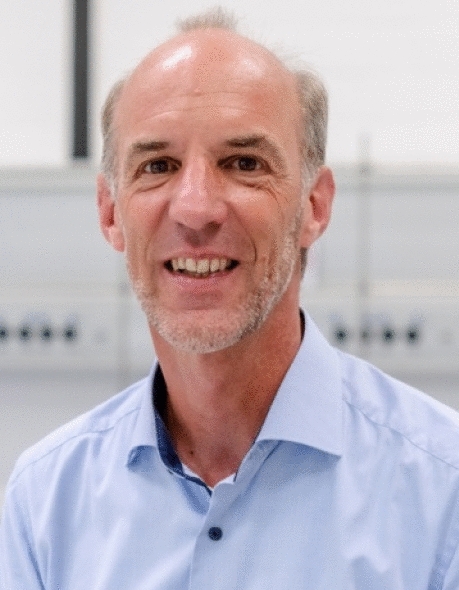# Editorial by the Chief Editor

**DOI:** 10.1007/s00775-025-02104-1

**Published:** 2025-02-27

**Authors:** Nils Metzler-Nolte

**Affiliations:** Faculty of Chemistry and Biochemistry, Chair of Inorganic Chemistry I – Bioinorganic Chemistry, Ruhr University Bochum, Bochum, Germany

Happy 30th Birthday JBIC!

This Editorial marks the first Issue of your favourite journal, JBIC, in the year of 2025 — and it is the 30th Volume of JBIC published! I take this anniversary as an opportunity to reflect on the past and future of publishing a Society journal.

**Fig. 1:**
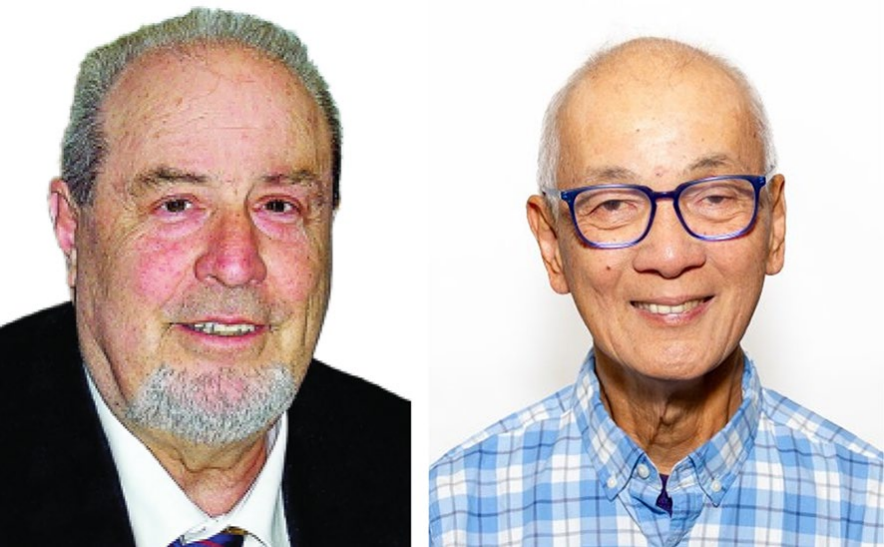
Prof. I. Bertini (left), founding Editor of JBIC and Prof. L. Que, Chief Editor of JBIC from 1999 – 2020 (right)

Thirty years ago, Bioinorganic Chemistry was a young, nascent field of research. It was not regarded as an established discipline in its own right, and articles with a “bioinorganic” topic were frequently rejected by traditional inorganic (“too much biology”) or biology (“inorganic / metal-based focus”) journals. To improve that situation, the Society of Biological Inorganic Chemistry (SBIC) was founded in 1995, along with its own journal, the Journal of Biological Inorganic Chemistry (JBIC). JBIC’s first Issue appeared in February 1996, with ten articles and kick-started by a minireview on iron–sulphur clusters [1]. Professor Ivano Bertini from Florence, Italy, acted as Founding Editor of JBIC. He was suceeded 3 years later by Professor Lawrence “Larry” Que (Minneapolis, USA), who guided the JBIC development through the following 20 years (Fig. 1).

Scientific publishing has undergone an enormous change since the early days of JBIC. In the 1990s, manuscripts were submitted as paper copies by postal mail (three copies!). Communication was again by exchanging (hand-)written letters, or using land-line telephone calls. More importantly, the choice of a journal was governed mostly by the topic area of the journal, its (perceived) prestige, and more often than not by personal relations (“I know the Editor well”).

Beginning in the 2000s, the entire process of submission, review, and publishing has completely transformed to a digitised world where not even copyright forms need “real” signatures any more, and all communication is electronic anyway. To the dismay of authors, many tasks that used to be “Editorial” are now carried out by the authors themselves. The creation of numerous new journals has rendered the publishing landscape virtually inextricable. And for many researchers, publication metrics govern the choice of a journal for their work more than anything else. At the same time, bibliometric analyses not only of single researchers (e.g. for appointment, tenure, and promotion) but even institutions and whole research fields assign an enormous impact to “publications” in a quantified, numerical way. For reasons beyond the scope of this Editorial, the fast, automated numerical, bibliometric analysis of journals has (largely, for most purposes) replaced the slow, careful analysis of a particular journal’s articles and their scientific content or even value.

Whilst those developments happened gradually over the last two decades, and probably mirror societal changes and altered human behaviour en large, JBIC has navigated well through those changes and has maintained its position as a premier journal for Bioinorganic Chemistry. Today, Bioinorganic Chemistry is an established interdisciplinary research field. “Bioinorganic” papers do not raise particular attention any more in broader inorganic, biochemical, biomedical or bioanalytical journals. Indeed for the most part, those papers contribute positively to their target journals’ prestige and not least to their metrics as well.

Now, as JBIC enters its fourth decade new challenges are rapidly approaching. Most importantly, the transformation of the “publishing market” to Open Access publishing, in a societal climate that demands more “open science”, essentially threatens to reverse the role of authors and journals. When formerly a journal’s reputation and appeal would dictate which articles were published (executed through — hopefully — responsible and insightful Editorial staff), it will more and more be the authors with their financial firepower who shape the publishing landscape and determine which journals will flourish. Equally disruptive is the advent of generative artificial intelligence (AI) and large language models (LLM). The use of those certainly has the power to make science more equitable and remove bias in publishing. On the dark side, misuse of these “tools” has created stacks of incorrect, fraudulent, and often outright fake papers. Publishers were largely caught off-guard and are struggling to regain control. Worse yet, the combination with the Open Access publishing model creates thoroughly undesirable incentives to lower scientific standards for submissions and barriers for acceptance since every single paper published now equates to direct income for the publisher. Indeed, the advent of a whole grey (if not outright black) market of “predatory publishers” could only develop in this unhealthy ecosystem of modern publishing.

What does this mean for our birthday child JBIC? It is my firm believe that the scientific community will need to push back on these unhealthy publishing practises. We must arrive at a less number-based approach to publishing. Scientific rigour, quality and competence in the publishing process, and credibility of journals will need to (again) be the deciding factors for our choice of publishing house and journal. In this sense, learned societies who own journals and guarantee the quality and integrity of their scientific content serve as an important safeguard against purely profit-oriented publishing. This is resembling the situation 30 years ago when the foundation of a scientific society was vital to putting a new topic on the scientific landscape, and its credibility was instrumental for establishing a now reputed journal in this (new) field. Today, when even top-level politicians display a disturbing disrespect for scientific results and data-based recommendations, there is a distinct role for learned societies and society-owned journals to guarantee scientific integrity and uphold trust in science. JBIC accepts that role, and will continue to serve our community in this spirit, as well as society in general. But it is also the individual duty of all members of our community to protect a trustworthy publication system. Every one of us must understand the dangerous, damaging logic behind „predatory publishing“. Stop submitting work to journals with doubtful reputation is a necessary first step, and one that can be implemented immediately! Also, we must teach our students to maintain highest academic standards and identify and boycott those who do not adhere to them. This includes investing more care into which papers we cite – relying on trusted sources and citing only those with a „quality stamp“ like being overseen by learned societies or otherwise trustworthy bodies is a necessary long-term measure to re-establish a publication landscape where quality, not money or quantity, rules. We rely on our community to help with this endeavour by submitting quality work to JBIC, cite articles in JBIC where appropriate, and thereby support scholarly publishing and science in a more general sense. Together, we will navigate JBIC through the upcoming challenges and changes, and I shall be happy to see a “grown-up” JBIC thrive in the coming years.

Nils Metzler-Nolte

January 2025